# Social Capital and Job Performance: A Moderated Mediation Model of Organizational Citizenship Behaviors and Psychological Capital

**DOI:** 10.3390/bs15060714

**Published:** 2025-05-22

**Authors:** Qi Cao, Chun-Fu Chen, Hui-Ling Hu, Yueh-Chih Hsiao

**Affiliations:** 1 Graduate School of International Studies, Hanyang University, Seoul 04763, Republic of Korea; qcaoae@connect.ust.hk; 2 Institute of Creative Design and Management, National Taipei University of Business, Taoyuan 324022, Taiwan; mark0617@ntub.edu.tw (C.-F.C.); huling0215@ntub.edu.tw (H.-L.H.); 3 Department of Technology Application and Human Resource Development, National Taiwan Normal University, Taipei 106308, Taiwan

**Keywords:** social capital, organizational citizenship behavior, psychological capital, job performance

## Abstract

Taiwan’s high-tech industry is experiencing rapid labor restructuring driven by automation and technological advancement, resulting in increased job demands and workplace stress. In this context, identifying mechanisms that sustain employee performance has become a critical concern. Drawing on the concept of social capital and grounded in Conservation of Resources (COR) theory, this study investigates whether social capital indirectly enhances job performance through organizational citizenship behavior. In addition, psychological capital is introduced as a moderating variable to examine the boundary conditions of this mediation process. Data were collected from 327 employees working in Taiwan’s high-tech sector and analyzed using PROCESS Model 14. The results indicate that social capital positively influences job performance only through the mediating effect of organizational citizenship behavior. Moreover, this indirect effect is strengthened when individuals possess higher levels of psychological capital. Based on these findings, the study concludes by discussing theoretical contributions and practical implications for organizations operating in high-pressure, innovation-driven environments.

## 1. Introduction

The high-tech sector in the Taiwan region, particularly in semiconductors, electronics, and information and communication technologies (ICT), has long served as a major economic driver (Rasiah et al., 2016; Suppipat & Hu, 2022). The region plays a pivotal role in the global semiconductor supply chain, accounting for 73% of the Asian foundry market and as much as 80% of the global market share (C. Liu et al., 2024). Globally renowned companies such as TSMC, MediaTek, and Foxconn anchor Taiwan’s strategic position in the high-tech supply chain (Hao & Bu, 2022). The ICT sector alone contributes over 80% of the region’s GDP, highlighting its central role in sustaining long-term economic growth (Lin & Park, 2023).

In addition to its contribution to GDP, the ICT sector also constitutes a significant portion of regional employment and exports. In recent years, electronics and semiconductor products have comprised more than 60% of Taiwan’s total exports (International Trade Administration, Ministry of Economic Affairs, 2025). Compared to other advanced economies in Asia, such as South Korea or Singapore, the Taiwan region holds a uniquely concentrated position in global semiconductor manufacturing.

However, despite its strategic importance, the high-tech sector is undergoing a profound transformation fueled by the rapid rise of artificial intelligence (AI) and automation technologies. These advancements are reshaping organizational structures, leading many firms to automate routine tasks, reduce labor reliance, and restructure workflows. Consequently, employees are now facing greater job insecurity, heavier workloads, and the need to continuously update their skills. Prolonged exposure to such high-pressure environments may increase psychological strain, reduce motivation, and negatively impact engagement, innovation, and operational stability (Choi, 2011; Khaw et al., 2023; Rafferty & Griffin, 2006; Shah et al., 2017; Vakola & Nikolaou, 2005). As such, identifying strategies to help employees maintain stable and high-quality job performance in the face of continuous organizational change has become a critical concern for both scholars and practitioners.

Job performance serves as a key metric for assessing employees’ contributions to achieving organizational goals. It reflects not only the efficiency and quality of individual task execution but also the extent to which employees engage in behaviors that support broader organizational functioning (Borman & Motowidlo, 1997). Prior research on job performance has predominantly focused on individual-level psychological and attitudinal factors. Job satisfaction remains one of the most extensively studied predictors, consistently demonstrating a positive relationship with performance outcomes (Iaffaldano & Muchinsky, 1985; Judge et al., 2001; Katebi et al., 2022). Other studies have highlighted the importance of work engagement and intrinsic motivation (Rich et al., 2010), as well as learning behaviors such as individual learning, team learning, and OCB (Atatsi et al., 2019).

In recent years, the role of interpersonal relationships within teams has become increasingly significant in determining performance outcomes (Clausen et al., 2019; Fonti & Maoret, 2016; Kerrissey & Novikov, 2024; Yen et al., 2020). Among the various factors affecting job performance, social capital has emerged as a vital resource that is embedded in interpersonal networks and enables the exchange of both tangible and intangible benefits through social interactions (Nahapiet & Ghoshal, 1998). Social capital fosters trust, shared goals, and mutual assistance, thereby improving individual and organizational efficiency (Andrews, 2010; Luthans et al., 2024; Sözbilir, 2018). However, the existing literature has predominantly focused on the direct relationship between social capital and job performance, with relatively limited attention given to how social capital may influence performance outcomes indirectly through employee behavioral mechanisms. In light of the growing emphasis on collaboration, knowledge sharing, and team-based structures in contemporary organizations, understanding the relationship between social capital and job performance has become increasingly salient. Accordingly, this study investigates how social capital influences employee job performance, with a particular focus on the behavioral mechanisms that may underlie this relationship.

Among various behavioral mechanisms, one of the most representative is organizational citizenship behavior (OCB). OCB has long been recognized as a critical construct in understanding how employees contribute to organizational effectiveness beyond their formal job responsibilities (Organ & Ryan, 1995). It encompasses a range of discretionary behaviors such as helping colleagues, demonstrating initiative, and exhibiting organizational loyalty that are essential for cultivating a cooperative work climate and reinforcing overall performance outcomes (Iqbal et al., 2022; Qalati et al., 2022; Zhang et al., 2022). As such, OCB serves as a particularly relevant behavioral pathway through which the effects of social capital on job performance may be channeled.

However, the relationship between social capital and OCB remains inconclusive in the existing literature, and theoretical explanations for this relationship are still lacking. Therefore, this study draws on the Conservation of Resources (COR) theory to re-examine the underlying mechanism linking the two constructs. According to Luthans et al. (2024) social capital refers to the network of relationships formed through interactions among organizational members and with external partners. These relationships foster collaboration and trust, thereby enhancing work efficiency. From the perspective of COR theory, social capital represents a type of social resource that can serve as a trigger for altruistic behaviors among employees, such as OCB. Accordingly, this study proposes that social capital can stimulate citizenship behaviors among organizational members, which in turn contribute to improved job performance.

Moreover, in today’s globally competitive landscape, while social capital facilitates the rapid establishment of stable relationships, it alone may not be sufficient to sustain long-term competitive advantages (Luthans et al., 2024). According to COR theory, individual characteristics can influence how individuals perceive the availability of resources, thereby amplifying the effects of others’ resource-depleting or resource-enriching behaviors (Halbesleben et al., 2014; Hobfoll et al., 2018). Psychological capital (PsyCap) is essential for achieving sustained competitive advantages at both the individual and organizational levels (Luthans et al., 2008; Vilarino del Castillo & Lopez-Zafra, 2022; Yao et al., 2022). Research has shown that employees with high PsyCap are more likely to engage in voluntary OCB (Avey et al., 2011; Kong et al., 2018; Wu & Nguyen, 2019), and exhibit superior job performance (Loghman et al., 2023). While prior studies have demonstrated that PsyCap can moderate relationships between leadership styles and employee behavior (Baig et al., 2021) as well as between emotions and workplace behaviors (Su & Hahn, 2023; Yao et al., 2022; Yildiz, 2019), whether PsyCap plays a moderating role in the OCB and job performance relationship remains an unexplored research question. Therefore, beyond examining the mediating process, it is worth further exploring whether the pathway through which social capital influences job performance via OCB may vary under different contextual conditions.

**Figure 1:**
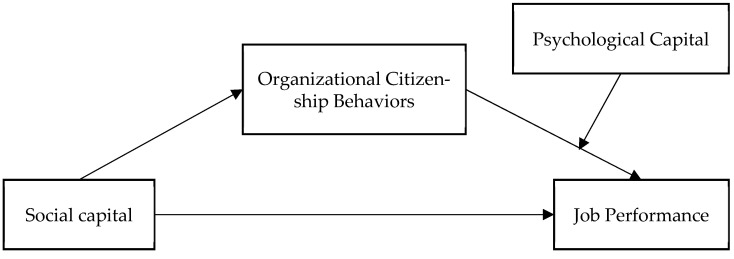
Research framework.

In summary, this study, grounded in COR theory, examines the relationships among social capital, OCB, and job performance, while verifying the mediating role of OCB. Additionally, this research investigates the moderating effect of PsyCap on the relationship between OCB and job performance and confirms the presence of a moderated mediation effect. Our theoretical model is presented in Figure 1. Based on the theoretical framework and empirical aims, this study seeks to address the following research questions:

RQ1: How does social capital influence job performance in high-tech organizational settings?

RQ2: Does OCB mediate the relationship between social capital and job performance?

RQ3: Does PsyCap moderate the effect of OCB on job performance, and does it condition the indirect effect of social capital through OCB?

## 2. Literature Review and Hypotheses Development

### 2.1. Conservation of Resource (COR) Theory

COR theory, proposed by Hobfoll (1989), is a resource-oriented framework that explains how individuals respond to external stressors and challenges. The theory posits that individuals actively seek to preserve their existing resources while simultaneously striving to acquire new ones to mitigate the adverse effects of stress (Hobfoll, 1989). It emphasizes the significance of resources in individual adaptability and psychological well-being, suggesting that resource depletion or threats to resources can lead to psychological stress, ultimately influencing behavior and decision-making (Hobfoll, 2001).

Hobfoll (2011) defines resources as tangible or psychological assets that individuals perceive as valuable, classifying them into four categories. Object resources refer to tangible assets that directly satisfy individual needs, such as money, equipment, and property. Condition resources encompass social roles, status, or environmental conditions that individuals possess, including job positions, marital status, and stable employment. Personal resources involve intrinsic psychological characteristics and capabilities, such as self-esteem, self-efficacy, and resilience. Lastly, energy resources serve as fundamental assets that facilitate the acquisition of other resources, including time, physical energy, and social capital (Hobfoll, 2011). These resources play a crucial role in helping individuals sustain their well-being, adapt to changing environments, and enhance their ability to cope with challenges.

Moreover, COR theory is built upon four core principles. The resource loss spiral suggests that when individuals experience resource depletion, they may enter a vicious cycle of continued resource loss, exacerbating their stress and diminishing their ability to function effectively (Halbesleben et al., 2014). Conversely, the resource gain spiral indicates that individuals with greater resource availability are more likely to accumulate additional resources, fostering a cycle of positive resource growth (Hobfoll, 2011; Hobfoll et al., 2018). The resource investment for protection principle states that individuals strategically invest their existing resources to safeguard or maintain their critical assets, reducing the negative impact of resource loss. Finally, the resource threat and stress reaction principle highlights that when individuals perceive their resources as being at risk of depletion, they experience psychological stress, which, in turn, influences their behaviors and decision-making (Halbesleben et al., 2014).

In recent years, COR theory has been widely applied in organizational behavior and workplace research. Studies have demonstrated that social capital, as a key resource, facilitates employees’ access to other valuable resources in the workplace (Clausen et al., 2019; Kerksieck et al., 2019). Additionally, social capital enhances mutual assistance behaviors among employees (Halbesleben & Wheeler, 2015), fosters trust within organizations, and ultimately contributes to improved job performance (Kidron & Vinarski-Peretz, 2024).

### 2.2. Social Capital and Job Performance

Social capital refers to the sum of actual and potential resources embedded within an individual’s social network that can be accessed or mobilized through interpersonal connections (Nahapiet & Ghoshal, 1998). In organizational contexts, social capital serves as a critical workplace resource that promotes employees’ work-related well-being, job engagement, and job performance (Clausen et al., 2019). Moreover, social capital encompasses both internal ties among employees and external ties with partners, clients, and other stakeholders (Carmeli et al., 2009; Xu et al., 2022). These relational networks facilitate the exchange of information, emotional support, and shared norms, thereby enhancing employees’ motivation and performance (Ben Hador, 2016; Luthans et al., 2024). Empirical studies have shown that mutual trust, reciprocity, and friendship among colleagues positively influence innovation, knowledge sharing, and overall organizational effectiveness (Andrews, 2010; Sözbilir, 2018).

From COR theory, individuals are motivated to acquire, preserve, and protect valuable resources to cope with stress and maintain performance in demanding environments (Hobfoll, 2011). These resources may be material, personal, or social, and their availability reduces the psychological and emotional costs of adaptation. Social capital, as a form of social resource embedded in interpersonal relationships, grants employees access to emotional support, instrumental assistance, and valuable information, key assets that help them navigate workplace challenges and maintain effective functioning (Halbesleben et al., 2014). Employees with higher levels of social capital are more likely to conserve and deploy their resources effectively, resulting in improved job performance. Therefore, we propose the following hypothesis:

**H1.**  *Social capital is positively related to job performance*.

### 2.3. Social Capital and Organizational Citizenship Behavior

Organizational Citizenship Behavior (OCB) refers to employees’ voluntary actions that go beyond formal job responsibilities to support organizational functioning and team collaboration (Organ & Ryan, 1995). Such behaviors include proactively assisting colleagues, actively participating in organizational activities, maintaining a harmonious workplace environment, and demonstrating a high sense of responsibility (Iqbal et al., 2022; Qalati et al., 2022; Zhang et al., 2022).

However, existing research has yet to reach a consensus regarding the relationship between social capital and OCB. Some scholars argue that OCB can strengthen social capital, as employees who engage in altruistic behaviors and proactive cooperation are more likely to develop strong social networks and trust-based relationships (Ariani, 2012; Basu et al., 2017; Bolino et al., 2002). Conversely, other studies suggest that social capital serves as a key determinant of OCB, asserting that individuals with abundant social capital—such as trust, reciprocity, and social connections—are more likely to exhibit OCB (Ko et al., 2018; Son et al., 2016; Yang et al., 2011). Given these divergent perspectives, further research is needed to clarify the relationship between social capital and OCB. To bridge this research gap, this study adopts COR theory to elucidate the link between social capital and OCB and further examines the mediating effect of OCB in the relationship between social capital and job performance.

From the perspective of COR theory, individuals’ initial resource endowments can accumulate and facilitate the generation of subsequent resources, thereby creating a positive resource spiral effect (Hobfoll, 2011; Hobfoll et al., 2018). The emotional support, information sharing, and reciprocity derived from social capital reinforce organizational cohesion and cooperation (Nahapiet & Ghoshal, 1998). Empirical studies have consistently demonstrated that social capital effectively promotes helping behaviors and OCB. For example, Yang et al. (2011), drawing from social exchange theory, found that trust and information exchange within social capital foster interpersonal helping behaviors. Similarly, Ko et al. (2018) provided empirical evidence that organizational members with abundant social capital are more willing to engage in OCB. Additionally, research on cyberbullying and misinformation has highlighted that social capital manifested through shared values and social trust encourages community members to exhibit civic behaviors that counteract bullying and misinformation (Son et al., 2016).

Building upon the aforementioned theoretical and empirical evidence, this study posits that individuals with rich social capital are more likely to cultivate shared identities and trust within their groups, thereby fostering reciprocal helping behaviors. Accordingly, we propose the following hypothesis:

**H2.**  *Social capital is positively related to organizational citizenship behavior*.

### 2.4. Organizational Citizenship Behavior and Job Performance

OCB plays a crucial role in enhancing both individual job performance and overall organizational effectiveness (N. P. Podsakoff et al., 2009; Zhang et al., 2022). Existing research has consistently demonstrated that OCB positively influences employees’ job performance (Chiang & Hsieh, 2012; Iqbal et al., 2022; Loghman et al., 2023; Qalati et al., 2022). Specifically, both helping-oriented OCB (e.g., altruism) and organization-focused OCB (e.g., civic virtue and conscientiousness) significantly contribute to improving employees’ job performance (Hermanto & Srimulyani, 2022). Furthermore, Gupta et al. (2024) integrating expectancy theory and social exchange theory, provided additional empirical evidence supporting the positive impact of OCB on job performance.

From the perspective of COR theory, this study posits that OCB can be regarded as a form of proactive resource investment in the workplace. Employees who voluntarily exert extra effort contribute to the organization’s operational efficiency, which, in turn, enhances their job performance. Accordingly, this study proposes the following hypothesis:

**H3.**  *Organizational citizenship behavior is positively related to job performance*.

### 2.5. The Mediation Role of Organizational Citizenship Behavior and Job Performance

Although previous studies have examined the relationships among social capital, OCB, and job performance, gaps remain in the existing literature. For instance, Ariani (2012), in a study involving 636 Indonesian banking employees, found that OCB enhances job performance through social capital. Similarly, Basu et al. (2017) focusing on healthcare employees, argued that the link between OCB and job performance is primarily mediated by social capital. However, these studies primarily rely on empirical findings and lack a comprehensive and systematic theoretical framework to explain the underlying mechanisms governing these relationships. Consequently, there remains a need for a more integrative theoretical explanation.

In contrast, this study extends previous empirical research by incorporating COR theory to provide a theoretical foundation for explaining these relationships. According to COR theory, individuals in the workplace acquire, maintain, and accumulate resources through social capital, which subsequently fosters the occurrence of OCB. The positive resource feedback mechanism generated by OCB further enhances job performance, ultimately creating a positive resource accumulation cycle, aligning with the resource gain spiral concept in COR theory (Hobfoll et al., 2018).

Prior research has shown that individuals with extensive and supportive networks, both within and outside the workplace, tend to exhibit greater care, empathy, and trust toward their network partners. This increased sense of social responsibility fosters OCB, which, in turn, enhances individual task performance (Bruque et al., 2016). Based on these theoretical and empirical foundations, this study proposes the following hypothesis:

**H4.**  *Organizational citizenship behavior mediates the positive relationship between social capital and job performance*.

### 2.6. The Moderation Role of Psychological Capital

Psychological capital (PsyCap), as a higher-order personal resource comprising self-efficacy, hope, resilience, and optimism (Luthans et al., 2007), plays a critical role in shaping how employees respond to workplace demands and opportunities. Beyond its direct effects, PsyCap functions as a psychological buffer and performance enhancer, particularly in high-pressure work environments (Luthans et al., 2024). Self-efficacy refers to an individual’s belief in their ability to successfully complete tasks and achieve goals. Hope reflects an individual’s capacity to generate multiple pathways to attain objectives while maintaining the motivation to overcome challenges. Resilience denotes an individual’s ability to adapt and recover when facing adversity, stress, or change. Optimism represents a positive outlook on the future, including confidence in one’s ability to influence outcomes (Luthans & Youssef-Morgan, 2017; Newman et al., 2014).

The relationship between PsyCap, OCB, and job performance has been well-documented in prior research. For instance, Avey et al. (2011) and Kong et al. (2018) confirmed through meta-analyses that individuals with abundant PsyCap exhibit significantly higher levels of OCB and job performance. Additionally, Wu and Nguyen (2019) not only verified the positive relationship between PsyCap and OCB but also demonstrated its positive association with job satisfaction and organizational commitment. Furthermore, Loghman et al. (2023) conducted a network meta-analysis to explore the antecedents, consequences, and moderating mechanisms of PsyCap. Their findings highlighted that PsyCap reduces job burnout and turnover intentions while enhancing job satisfaction, work engagement, and job performance.

Beyond its direct effects, PsyCap functions as a crucial personal psychological resource that moderates workplace attitudes and behaviors. For example, Yildiz (2019) found that positive PsyCap enhances the relationship between organizational trust and OCB, suggesting that employees with higher PsyCap are more likely to engage in discretionary behaviors beneficial to organizational functioning when they perceive strong organizational trust. Similarly, Yao et al. (2022) found that PsyCap strengthens the relationship between work engagement and job performance.

In a systematic literature review, Vilarino del Castillo and Lopez-Zafra (2022) concluded that PsyCap most commonly serves as a moderator in relationships involving creativity, psychological well-being, job performance, and job satisfaction. Baig et al. (2021) also provided empirical evidence that PsyCap positively moderates the relationship between leadership style and job performance. Their findings indicate that employees with higher levels of PsyCap respond more effectively to both transformational and transactional leadership, resulting in enhanced performance outcomes. More recently, research has begun to explore PsyCap’s moderating role in mechanisms related to OCB. For example, Su and Hahn (2023) demonstrated that high levels of PsyCap facilitate resource preservation and development, thereby motivating employees to engage in OCB. Collectively, these findings highlight PsyCap’s potential to strengthen the link between positive employee behaviors and favorable job outcomes.

From the perspective of COR theory, internal psychological resources such as PsyCap enable employees to buffer resource depletion and enhance the utilization of external or behavioral resources, including OCB. PsyCap, comprising self-efficacy, hope, resilience, and optimism, supports individuals in sustaining motivation, coping with adversity, and recovering from setbacks. These traits empower employees to persist in discretionary efforts like OCB and to translate such behaviors into tangible performance outcomes (Avey et al., 2011; Halbesleben et al., 2014; Luthans & Youssef-Morgan, 2017).

Accordingly, this study posits that PsyCap moderates the relationship between OCB and job performance. Employees with higher levels of PsyCap are more likely to maximize the benefits of their citizenship behaviors by maintaining a positive work mindset and effectively navigating workplace challenges. In contrast, those with lower PsyCap may lack the psychological resources to convert OCB into improved performance. Based on this reasoning, the following hypothesis is proposed:

**H5.**  *Psychological capital moderates the relationship between organizational citizenship behavior and job performance, such that the positive relationship is stronger when psychological capital is high*.

According to Preacher et al. (2007), moderated mediation occurs when a moderator variable not only influences the strength of the relationship between an independent variable and a mediator but also alters the magnitude of the indirect effect from the independent variable to the dependent variable through the mediator. This conceptual framework provides a foundation for understanding how psychological or contextual factors can shape the effectiveness of indirect mechanisms in organizational behavior.

Drawing on this perspective, and grounded in COR theory, this study proposes that PsyCap strengthens the indirect effect of social capital on job performance through OCB. PsyCap, comprising hope, efficacy, resilience, and optimism, functions as a personal resource that facilitates adaptive, goal-directed behavior under pressure (Luthans & Youssef-Morgan, 2017; Newman et al., 2014). Empirical studies have shown that individuals with higher PsyCap have more sustained OCB and job performance (Avey et al., 2011; Kong et al., 2018; Loghman et al., 2023; Wu & Nguyen, 2019). Based on these findings, it is reasonable to infer that employees with higher PsyCap can more effectively leverage social capital, such as trust and collaboration, to engage in OCB, which in turn promotes higher performance. Accordingly, this study adopts a moderated mediation model in which PsyCap not only moderates the direct relationship between OCB and job performance but also amplifies the indirect effect of social capital through OCB. Based on this reasoning, this study proposes the following hypothesis:

**H6.**  *Psychological capital moderates the mediating effect of organizational citizenship behavior between social capital and job performance, such that the indirect relationship is stronger when psychological capital is high and weaker when psychological capital is low*.

## 3. Research Methodology

### 3.1. Participants and Procedure

This study employed a purposive sampling method targeting employees in Taiwan’s high-tech industry. Participants were drawn from technology companies across sectors such as semiconductors, electronics, and information services. The sampling strategy aimed to capture individuals working in environments characterized by rapid innovation, high performance demands, and cross-functional collaboration conditions that are theoretically relevant to the constructs under investigation.

To ensure data relevance and quality, the inclusion criteria required participants to be full-time employees with a minimum of three years of work experience and active involvement in team-based tasks. The questionnaire was distributed electronically with the assistance of HR professionals from the researchers’ personal network, who helped forward the survey to eligible employees within their organizations.

To mitigate common method variance (CMV), this study followed the recommendations of P. M. Podsakoff et al. (2003) and adopted several procedural remedies. These included anonymizing item meanings and randomizing the order of questionnaire items to prevent response patterns. Such techniques were implemented to reduce potential response biases and enhance the validity and reliability of the collected data.

Furthermore, to ensure clarity, validity, and appropriateness, the questionnaire underwent expert validation by three academic specialists before distribution. Their feedback was incorporated to refine the items, ensuring that the measurement instruments accurately captured the intended constructs. Following this validation process, the finalized questionnaire was administered for data collection. A total of 442 responses were received; however, after excluding incomplete or inconsistent responses, a final sample of 327 valid questionnaires was retained for subsequent statistical analysis. This sample size meets the recommended threshold of at least 200 participants, as suggested by (Barrett, 2007) ensuring the robustness and generalizability of the study’s findings.

The sample characteristics indicate that the majority of participants were male (85.6%). In terms of work experience (seniority), 29.4% of respondents had less than five years of experience, 34.6% had between six and ten years, and 36.1% had more than ten years of experience. Concerning educational background, 10.7% of participants had a high school education or below, 78.0% held a bachelor’s degree, and 11.3% possessed a graduate degree or higher (Table 1).

### 3.2. Questionnaire Design

This study employed a seven-point Likert scale for questionnaire design, with response options ranging from “strongly disagree” (1) to “strongly agree” (7). Measurement instruments were adapted from the established literature and refined to align with the study’s context, ensuring scale validity and applicability.

#### 3.2.1. Social Capital

The social capital scale, adapted from C.-H. Liu and Jiang (2020), consists of four items assessing respondents’ interactions, knowledge-sharing behaviors, and learning experiences within their organization. Sample items included: “I am skilled at collaborating with my colleagues in a team” and “I can interact and exchange ideas with individuals from different fields”. Reliability analysis indicated a Cronbach’s α of 0.881, demonstrating good internal consistency.

#### 3.2.2. Organizational Citizenship Behavior (OCB)

The OCB scale, based on Lee and Allen (2002), consists of five items measuring employees’ willingness to engage in altruistic behaviors in the workplace. Representative items included the following: “I am willing to take time to help colleagues solve work-related problems” and “I assist my colleagues in completing their tasks”. The scale exhibited strong reliability, with a Cronbach’s α of 0.927.

#### 3.2.3. Psychological Capital (PsyCap)

The PsyCap scale was measured using the short version of the Psychological Capital Questionnaire (PCQ-12) developed by Luthans et al. (2007). This instrument assesses four dimensions—self-efficacy, hope, resilience, and optimism—each measured with three items, for a total of 12 items. A second-order confirmatory factor analysis (CFA) confirmed the scale’s structure, with standardized factor loadings ranging from 0.613 to 0.953 and no items removed. Model fit indices were acceptable: χ^2^ = 286.321, *p* < 0.001, CFI = 0.894, NFI = 0.874, TLI = 0.868, IFI = 0.895, and SRMR = 0.073. The overall Cronbach’s α was 0.910, indicating strong reliability (Hu & Bentler, 1999). The detailed items of psychological capital are presented in Appendix A.

#### 3.2.4. Job Performance

The job performance scale, adapted from Pitafi et al. (2018), includes six items measuring self-perceived job performance. Sample statements included the following: “I always put in extra effort at work” and “I deliberately invest additional energy into my job”. The scale demonstrated strong internal reliability, with a Cronbach’s α of 0.892.

### 3.3. Common Method Variance

In light of the potential concern regarding common method variance (CMV), which may arise due to the study’s reliance on self-reported data and the cross-sectional nature of data collection, rigorous methodological precautions were undertaken. Specifically, the study adhered to the procedural and statistical recommendations outlined by P. M. Podsakoff et al. (2003) to mitigate CMV-related biases. To empirically assess the extent of CMV, Harman’s single-factor test was performed. This involved subjecting all measurement items related to social capital, OCB, PsyCap, and job performance to an exploratory factor analysis (EFA) under a single-factor model. The results indicated that the unrotated factor solution accounted for 46.641% of the total variance, which remains below the widely accepted threshold of 50%. Consequently, these findings suggest that CMV is unlikely to pose a significant threat to the validity of the study’s conclusions.

### 3.4. Data Analysis

This study utilized AMOS 24.0 statistical software to conduct a confirmatory factor analysis (CFA) to assess the convergent and discriminant validity of the measurement scales, ensuring measurement validity and reliability. To further evaluate model fit and theoretical robustness, SPSS PROCESS version 4.2 was employed for hypothesis testing, incorporating path analysis, mediation analysis, moderation analysis, and moderated mediation analysis.

Following the recommendations of Preacher and Hayes (2008), a bootstrapping procedure with 2000 resamples was applied to estimate confidence intervals (CIs) and assess the significance of the hypotheses. Hypotheses were supported when the confidence interval exceeded zero, indicating a statistically significant effect. Conversely, if it did not, this showed that the hypothesis was not supported.

## 4. Research Results

### 4.1. Convergent Validity

Given the large number of measurement items in the PsyCap scale, there was a risk of underestimating overall model fit, potentially leading to poorer model fit indices (Nasser & Takahashi, 2003). To address this issue, Coffman and MacCallum (2005) recommended the item parceling method, which reduces the number of measurement indicators for each construct. This approach increases degrees of freedom and minimizes measurement error in CFA models (Williams & O’Boyle, 2008). Accordingly, this study adopted item parceling to enhance the robustness of the measurement model and ensure a more accurate assessment of construct validity.

As previously mentioned, the second-order CFA results for PsyCap indicated that all items demonstrated strong factor loadings with their respective dimensions. Therefore, following the approach suggested by Landis et al. (2000), this study combined items belonging to the same dimension of PsyCap, resulting in four parceled indicators representing the four PsyCap dimensions: self-efficacy, hope, resilience, and optimism. These parcels were subsequently used in the CFA model to assess the measurement structure.

As shown in Table 2, the factor loadings of all research variables exceeded 0.6 (Hair et al., 2019), indicating that the items effectively captured the underlying constructs. Furthermore, the composite reliability (CR) values were all above 0.7, demonstrating internal consistency, while the average variance extracted (AVE) values were greater than 0.5 (Fornell & Larcker, 1981), confirming good convergent validity.

### 4.2. Discriminant Validity and Correlation Analysis

The means, standard deviations, and correlation coefficients for all study variables are presented in Table 3. To assess discriminant validity, this study employed the Heterotrait–Monotrait Ratio (HTMT) criterion. The HTMT values ranged from 0.660 to 0.832, all below the recommended threshold of 0.85 (Hair et al., 2019), indicating that social capital, OCB, PsyCap, and job performance exhibited adequate discriminant validity at the theoretical level.

Furthermore, the correlation coefficients revealed a significant positive relationship between social capital and OCB (*r* = 0.699, *p* < 0.001), as well as between OCB and job performance (*r* = 0.623, *p* < 0.001). These results preliminarily align with the hypothesized relationships in this study. However, given the relatively high correlation coefficients among the variables, multicollinearity diagnostics were conducted to ensure robustness.

First, the variance inflation factor (VIF) values ranged from 2.169 to 2.573, all below the threshold of 5 (Hair et al., 2019). Additionally, the condition index was 26.932, which is below the recommended threshold of 30 (Draper & Smith, 1998). These results confirm that multicollinearity was not a concern in this study.

### 4.3. Path Analysis and Mediation Analysis

Before testing the hypotheses, a model fit analysis was conducted to ensure the appropriateness of the structural model. The model fit indices met recommended thresholds (χ^2^ = 473.662, *p* < 0.001, CFI = 0.929, NFI = 0.901, TLI = 0.917, IFI = 0.929, SRMR = 0.048, and RMSEA = 0.083), indicating an acceptable model fit (Hu & Bentler, 1999). The measurement model demonstrated satisfactory reliability and validity, confirming its suitability for subsequent hypothesis testing.

**Figure 2:**
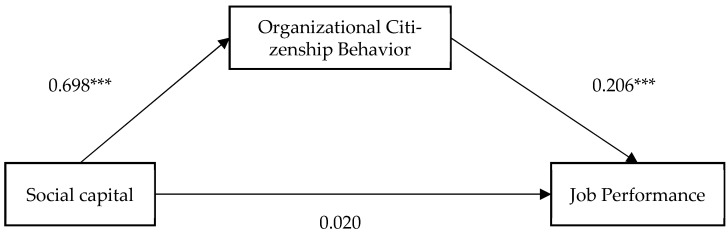
Path analysis results. *** *p* < 0.001.

This study employed SPSS PROCESS Model 14 to test all hypotheses. The results of the path analysis are presented in Table 4 and illustrated in Figure 2. The findings reveal that social capital has a significant positive effect on OCB (Coefficient = 0.698, *p* < 0.001), explaining 48.9% of its variance (Adjusted R^2^ = 0.489), thereby highlighting the importance of social capital in promoting OCB.

In addition, OCB positively and significantly affects job performance (Coefficient = 0.206, *p* < 0.001), underscoring its critical role in enhancing individual performance. However, the direct effect of social capital on job performance was not statistically significant (Coefficient = 0.020, *p* > 0.05), suggesting that social capital does not directly influence job performance. Social capital and OCB accounted for 58.7% of the variance in job performance (Adjusted R^2^ = 0.587). Based on the results, Hypothesis H1 was not supported, whereas Hypotheses H2 and H3 were supported.

This finding underscores the mediating role of OCB as a critical mechanism linking social capital to job performance. This study further examined the potential mediating role of OCB. The mediation analysis revealed a significant indirect effect of social capital on job performance through OCB (Coefficient = 0.144, SE = 0.033, z = 4.363, 95% CI [0.079, 0.215]). Since the confidence interval did not include zero, this result provides empirical support for Hypothesis H4.

In summary, social capital does not exert a significant direct effect on job performance. Instead, its influence is fully mediated by OCB. This finding suggests that employees with higher levels of social capital are more likely to exhibit discretionary behaviors that support the organization, which in turn enhance their job performance. The full mediation highlights the pivotal role of OCB as the key mechanism linking social capital to job performance. Consequently, fostering a collaborative and supportive work environment not only facilitates interpersonal interactions and knowledge sharing but also promotes OCB, ultimately leading to improved performance outcomes.

### 4.4. Moderation Analysis

**Figure 3:**
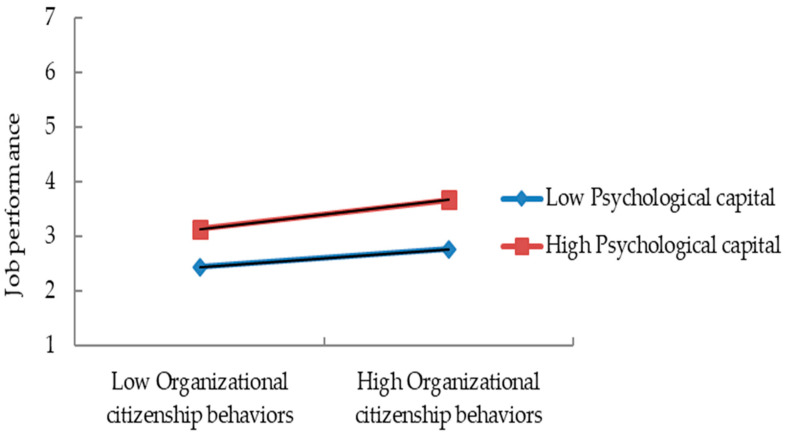
Simple slope analysis results.

To address potential multicollinearity concerns, both OCB and PsyCap were mean-centered prior to analysis, in accordance with the recommendations of Aiken and West (1991). The results revealed a significant interaction effect, indicating that PsyCap moderates the relationship between OCB and job performance (Coefficient = 0.052, SE = 0.023, z = 2.283, 95% CI [0.006, 0.097]). Moreover, the results of the simple slope test revealed that when PsyCap was high (mean +1 SD), the positive relationship between OCB and job performance was significant (Coefficient = 0.258, SE = 0.049, z = 5.258, 95% CI [0.161, 0.355]). Similarly, when PsyCap was low (mean −1 SD), the positive relationship between OCB and job performance also remained significant (Coefficient = 0.154, SE = 0.043, z = 3.531, 95% CI [0.068, 0.240]). Since the confidence interval did not include zero, this confirms that the moderating effect is statistically significant. Figure 3 illustrates the nature of this moderation effect, demonstrating that higher levels of PsyCap strengthen the positive relationship between OCB and job performance. Therefore, Hypothesis H5 is supported.

### 4.5. Moderated Mediation Model

As shown in Table 5, the results of the moderated mediation analysis revealed that when PsyCap was low (−1 SD), the indirect effect of social capital on job performance via OCB was 0.107 (Boot SE = 0.037, 95% CI [0.037, 0.190]). When PsyCap was at the mean level, the indirect effect increased to 0.144 (Boot SE = 0.033, 95% CI [0.079, 0.215]). Furthermore, when PsyCap was high (+1 SD), the indirect effect further increased to 0.180 (Boot SE = 0.036, 95% CI [0.108, 0.252]). In addition, the index of moderated mediation was 0.036 (Boot SE = 0.016, 95% CI [0.003, 0.067]), indicating that the conditional indirect effect significantly varied as a function of PsyCap.

In summary, these findings confirm that as PsyCap increases, the positive indirect effect of social capital on job performance through OCB is amplified. Therefore, Hypothesis H6 is supported.

## 5. Conclusions

This study, grounded in COR theory, systematically examines how social capital enhances job performance through OCB. In addition, it explores a moderated mediation model in which PsyCap moderates the indirect relationship between social capital and job performance via OCB such that the mediating effect of OCB becomes stronger when PsyCap is higher. Through a comprehensive model and empirical analysis, this research deepens our understanding of organizational behavior and extends the application of COR theory to explain performance enhancement mechanisms within the workplace.

First, the empirical findings confirm the significant relationship among social capital, OCB, and job performance, demonstrating that social capital positively influences job performance through OCB. This result aligns with the core tenets of COR theory, which suggest that individuals’ initial resources can be transformed into higher-level outcomes through positive interactions and resource accumulation (Hobfoll, 2011; Hobfoll et al., 2018), ultimately enhancing overall work performance. The findings are also consistent with those of Bruque et al. (2016), further substantiating the critical role of social capital in workplace environments.

However, the present study did not find a significant direct effect of social capital on job performance. This finding contrasts with several previous studies (Andrews, 2010; Ben Hador, 2016; Clausen et al., 2019; Sözbilir, 2018) that have reported a positive direct relationship between social capital and performance outcomes. The results suggest that social capital in the workplace does not directly enhance job performance; rather, it must be enacted through concrete cooperative behaviors to generate meaningful performance improvements. Accordingly, this study underscores the mediating role of OCB as a crucial behavioral mechanism through which social capital is transformed into tangible performance outcomes. Employees with abundant social capital are more likely to establish trust and a collaborative climate within the organization, thereby fostering higher levels of OCB—such as assisting colleagues, taking on additional responsibilities, and maintaining team harmony—which, in turn, contribute to enhanced job performance.

Second, this study identifies PsyCap as a crucial moderating factor in the relationship between OCB and job performance, revealing its positive moderation effect. Specifically, employees with higher PsyCap experience a stronger positive impact of OCB on job performance. This finding echoes COR’s resource spiral effect (Halbesleben et al., 2014; Hobfoll et al., 2018) suggesting that when individuals possess high levels of PsyCap, such as confidence, optimism, hope, and resilience, they are more likely to engage in workplace interactions proactively and display behaviors beneficial to the organization. These behaviors include helping colleagues, fostering a positive organizational climate, and assuming extra responsibilities (Avey et al., 2011; Hobfoll et al., 2018; Kong et al., 2018; Wu & Nguyen, 2019). Not only do these behaviors enhance employees’ perceived workplace value but they also contribute to the accumulation of additional work resources, reinforcing subsequent resource cycles and ultimately improving job performance (Loghman et al., 2023).

Finally, this study further validates the moderated mediation role of PsyCap, demonstrating that when PsyCap is high, the indirect effect of social capital on job performance through OCB becomes more pronounced. This finding suggests that PsyCap not only serves as a critical internal mechanism for resource accumulation but also amplifies resource conversion processes in workplace environments. This result is consistent with the resource enhancement process emphasized by COR theory (Halbesleben et al., 2014; Hobfoll, 2011; Hobfoll et al., 2018), which posits that individuals with higher PsyCap can more effectively transform external social capital into behavioral motivation, leading to greater engagement in OCB and ultimately strengthening job performance.

### 5.1. Theoretical Implications

The first theoretical contribution of this study lies in its application of COR theory to offer a novel perspective on the role of social capital in workplace interactions. This study conceptualizes social capital as an initial resource that individuals possess in the workplace, which helps shape positive interactions and trust-based relationships, ultimately motivating employees to engage in OCB. In other words, this research highlights that the occurrence of OCB is not solely the result of individual voluntary choices but is also driven by the social network environment in which the individual is embedded. Employees with abundant social capital, such as strong social networks and mutual trust, are more likely to exhibit altruistic behaviors beyond their formal job responsibilities, fostering a collaborative climate and enhancing organizational effectiveness. This perspective not only reinterprets the causal relationship between social capital and OCB but also expands the application of COR theory in the field of organizational behavior, addressing the research gap concerning the role of social capital as an antecedent of OCB.

The second theoretical contribution of this study lies in its systematic application of COR theory to explain how individuals can leverage their existing resources (social capital) to generate positive behaviors (OCB), which, in turn, create a cycle of resource accumulation and performance enhancement. Importantly, the findings revealed that social capital does not exert a direct effect on job performance, thereby highlighting the pivotal mediating role of OCB in translating social capital into tangible performance outcomes. This underscores the importance of behavioral mechanisms in the resource conversion process and affirms the value of OCB as a key conduit through which resources yield outcomes.

Furthermore, the results provide empirical support for the resource gain spiral proposed by COR theory, demonstrating how initial resources (social capital) can stimulate resource-generating behaviors (OCB), which then lead to enhanced job performance, thus reinforcing the cycle of resource enrichment. By emphasizing the dynamic and recursive nature of resource transformation, this study offers new insights into how social capital functions as a foundational resource that fosters proactive workplace behaviors and drives sustained performance improvements.

The third theoretical contribution of this study addresses a critical gap in the existing literature by illuminating the dynamic interplay between internal personal resources and workplace behaviors in shaping job performance. While previous research has often conceptualized resources as static and independent predictors, this study advances COR theory by demonstrating that internal resources (PsyCap) and behavioral expressions (OCB) interact continuously and reciprocally to influence performance outcomes. Drawing on the resource gain spiral effect proposed by COR theory (Halbesleben et al., 2014; Hobfoll, 2011; Hobfoll et al., 2018), the findings indicate that employees with higher levels of PsyCap are more likely to engage in OCB (Avey et al., 2011; Kong et al., 2018; Wu & Nguyen, 2019). Moreover, moderated mediation analysis revealed that PsyCap amplifies the indirect effect of social capital on job performance via OCB. Specifically, the mediating role of OCB becomes more pronounced when employees possess higher levels of PsyCap, thereby reinforcing the positive cycle of resource accumulation. This finding underscores the synergistic interaction between internal and external resources and supports the notion that the accumulation of resources is not a linear or static process but rather a dynamic and recursive system of mutual reinforcement.

### 5.2. Managerial Implications

Amid ongoing restructuring and workforce optimization in Taiwan’s high-tech sector, employees face heightened workloads, job insecurity, and increased stress. In such contexts, fostering social capital is essential for sustaining collaboration, knowledge exchange, and overall performance. Managers can take concrete actions to strengthen social capital by initiating cross-departmental projects, hosting monthly technical exchange workshops, and establishing formal mentorship programs that pair experienced employees with new hires. These strategies help embed trust and support into the organizational fabric while building robust internal networks.

Given the critical role of teamwork in R&D and production workflows, managers should implement team-based performance incentives, such as bonus systems tied to collaborative success, peer-nominated awards, and visible recognition events during company meetings to reinforce OCB behaviors. To maintain long-term behavioral consistency, organizations can adopt knowledge-sharing platforms (e.g., internal wikis or digital forums) and hold quarterly feedback sessions to encourage continuous engagement.

As the industry navigates digital transformation, PsyCap becomes a key resource in helping employees remain resilient and adaptable. To build self-efficacy, firms should offer technical upskilling programs, cross-functional job shadowing, and certification-based training paths that equip employees with tools for role expansion. Mentorship structures not only support learning but also create psychological safety during role transitions.

Cultivating optimism and hope can be achieved through transparent communication strategies, such as regular town hall updates, and career development planning, including internal mobility pathways and personalized growth plans. Employees who see a clear future within the organization are more likely to stay engaged and proactive. Finally, to foster resilience at the team level, managers can implement weekly strategic huddles, peer-led coaching groups, and collaborative innovation sprints, all of which enhance adaptability and cohesion during periods of organizational change.

### 5.3. Research Limitations and Future Research Developments

Grounded in COR theory, this study examined how social capital enhances job performance through OCB and further tested the moderated mediation effect of PsyCap. Despite its theoretical and empirical contributions, the study has several limitations that the authors of future studies could address to improve the robustness and generalizability of the findings.

First, this study’s sample consisted exclusively of employees from Taiwan’s high-tech industry, which may limit the generalizability of the findings to other cultural and industrial contexts. Work practices, organizational structures, and employee expectations within Taiwan’s technology sector may differ from those in other regions or sectors. Consequently, caution is warranted when applying these results to broader populations.

Furthermore, the study did not collect firm-level background data such as company size, ownership type, or industry sub-sector. The absence of these organizational characteristics limits the ability to assess whether firm-level variables may moderate the relationships among social capital, OCB, and job performance. As organizational structure and ownership models may influence employee behavior and workplace dynamics, the authors of future studies are encouraged to gather and analyze such contextual variables. This would allow for a more nuanced understanding of how individual-level relationships operate across different types of organizations and business environments.

Second, the study employed purposive sampling through the researchers’ professional network. While this approach enabled access to qualified respondents, it may also introduce selection bias. Although efforts were made to include participants from various job roles and company types, the non-random sampling method limits the external validity. The authors of future studies could adopt stratified or random sampling techniques and expand the sample to include broader demographics to improve representativeness.

Third, this study relied on self-reported data collected at a single point in time, which may raise concerns about common method variance (CMV). Although procedural remedies—such as item randomization and response anonymity—were applied and Harman’s single-factor test indicated no significant CMV, the risk cannot be fully eliminated. To reduce CMV risks, future studies could employ time-lagged designs or use multi-source data, such as supervisor-rated OCB and job performance, to enhance objectivity and mitigate common source bias. Combining self-reported data with supervisor assessments would provide a more comprehensive and accurate evaluation, thereby minimizing potential biases inherent in self-report methods.

Furthermore, to establish a clearer causal relationship between social capital and OCB, the authors of future studies could consider adopting experimental designs. For instance, researchers could manipulate levels of social capital within controlled settings (e.g., through team-building activities or social interaction interventions) and subsequently measure changes in OCB. Such an approach would allow for a more rigorous examination of causality, minimizing the limitations inherent in cross-sectional survey methods. By integrating experimental methods with observational data, it would be possible to achieve a more robust understanding of how social capital influences OCB.

Finally, while COR theory categorizes resources into object, condition, personal, and energy resources, this study primarily focused on personal resources—namely, social capital and PsyCap. However, object resources (e.g., compensation and access to advanced equipment) and condition resources (e.g., job security and organizational support) may also significantly shape employee behavior and performance. The authors of future studies should explore how these additional resource types interact with personal resources to influence OCB and job performance. Investigating potential synergistic effects between resource types may further enrich theoretical understanding and provide more nuanced managerial insights into resource dynamics in the workplace.

## Figures and Tables

**Table 1:** Profile of survey respondents (*n* = 327).

Characteristics	Classification	Frequency	Proportion (%)
Gender	Male	280	85.6
	Female	47	14.4
Seniority	Below 5	96	29.4
	6–10	113	34.6
	Above 10	118	36.1
Education	High school or below	35	10.7
	Bachelor’s degree	255	78.0
	Above a graduate degree	37	11.3

**Table 2:** Measurement model.

Factors and Items	Factor Loading	CR	AVE
Social Capital (SOC)			
1. I am good at collaborating with my colleagues.	0.791	0.888	0.657
2. I actively share information and engage in mutual learning with my coworkers.	0.885		
3. I interact and exchange ideas with people from different departments or fields.	0.747		
4. I am willing to collaborate with colleagues to solve problems together.	0.812		
Organizational Citizenship Behavior (OCB)			
1. I am willing to spend time helping coworkers solve work-related problems.	0.839	0.929	0.722
2. I assist junior colleagues in adapting to the organization.	0.821		
3. I sincerely care about the well-being of my coworkers.	0.893		
4. I am willing to spend time helping coworkers with personal challenges.	0.841		
5. I help my colleagues complete their tasks when needed.	0.852		
Psychological Capital (PsyCap)			
1. Self-efficacy	0.661	0.865	0.610
2. Hope	0.849		
3. Resilience	0.854		
4. Optimism	0.761		
Job Performance (JOP)			
1. My job performance is consistently above average.	0.702	0.886	0.578
2. My job performance often exceeds expectations.	0.705		
3. I always put in extra effort in my work.	0.756		
4. I invest a great deal of energy in my work.	0.798		
5. I always do my best in completing my tasks.	0.813		
6. The quality of my work is consistently high.	0.773		

**Table 3:** Discriminant validity analysis.

Variable	*M*	*SD*	SOC	OCB	PsyCap	JOP
Seniority	2.070	0.807	0.073	0.065	0.178 **	0.251 ***
SOC	5.454	0.793	-	0.699 ***	0.725 ***	0.589 ***
OCB	5.446	0.821	0.772	-	0.661 ***	0.623 ***
PsyCap	5.086	0.726	0.830	0.742	-	0.724 ***
JOP	5.087	0.757	0.660	0.669	0.832	-

Note: The lower triangular matrix presents the Heterotrait–Monotrait Ratio of Correlations (HTMT). Note: The upper triangular matrix presents the correlation coefficients. Note: SOC = social capital, OCB = organizational citizenship behavior, PsyCap = psychological capital, and JOP = job performance. *** *p* < 0.001; ** *p* < 0.01.

**Table 4:** Path analysis table.

Path Relationship	Coefficient	*SE*	*z*	95% CI
SOC → OCB	0.698	0.039	17.537 ***	[0.619, 0.776]
SOC → JOP	0.020	0.043	0.460	[−0.065, 0.106]
OCB → JOP	0.206	0.040	5.114 ***	[0.127, 0.286]
SOC → OCB → JOP	0.144	0.033	4.363 ***	[0.079, 0.215]

Note: SOC = social capital, OCB = organizational citizenship behavior, and JOP = job performance. *** *p* < 0.001.

**Table 5:** Moderated mediation model table.

Psychological Capital	Coefficient	*SE*	*z*	95% CI
−1 SD	0.107	0.037	2.891 **	[0.037, 0.190]
M	0.144	0.033	4.363 ***	[0.079, 0.215]
+1 SD	0.180	0.036	5.000 ***	[0.108, 0.252]
Index of MoMe	0.036	0.016	2.250 *	[0.003, 0.067]

*** *p* < 0.001; ** *p* < 0.01; * *p* < 0.05.

## Data Availability

The data presented in this study are available from the corresponding author upon request.

## Appendix A

Psychological Capital Scale (each dimension consists of three items)

Self-Efficacy

1. I am confident in representing my department in meetings with upper management.

2. I am confident in offering suggestions during team meetings.

3. I am confident in helping my team set work goals.

Hope

1. When faced with an excessive workload, I find ways to overcome it.

2. I consider myself to be quite successful in my work.

3. I try every possible method to achieve my work goals.

Resilience

1. I have been able to accomplish the work goals I set for myself.

2. I try to complete tasks independently whenever possible.

3. I am able to handle work-related pressure calmly.

Optimism

1. I always try to see the positive side of work situations.

2. I remain optimistic when facing challenges at work.

3. No matter how difficult the task, I believe there will be a positive outcome.
